# The Pharmacokinetic-Pharmacodynamic Model of Azithromycin for Lipopolysaccharide-Induced Depressive-Like Behavior in Mice

**DOI:** 10.1371/journal.pone.0054981

**Published:** 2013-01-24

**Authors:** Kun Hao, Qu Qi, Haiping Hao, Guangji Wang, Yuancheng Chen, Yan Liang, Lin Xie

**Affiliations:** 1 State Key Laboratory of Natural Medicines, Key Lab of Drug Metabolism & Pharmacokinetics, China Pharmaceutical University, Nanjing, China; 2 Institute of Antibiotics, Huashan Hospital, Fudan Univeristy, Shanghai, China; Emory University, United States of America

## Abstract

A mechanism-based model was developed to describe the time course of lipopolysaccharide-induced depressive-like behavior and azithromycin pharmacodynamics in mice. The lipopolysaccharide-induced disease progression was monitored by lipopolysaccharide, proinflammatory cytokines, and kynrenine concentration in plasma. The depressive-like behavior was investigated by forced swimming test and tail suspension test. Azithromycin was selected to inhibit the surge of proinflammatory cytokines induced by lipopolysaccharide. Disease progression model and azithromycin pharmacodynamics were constructed from transduction and indirect response models. A delay in the onset of increased proinflammatory cytokines, kynrenine, and behavior test compared to lipopolysaccharide was successfully characterized by series transduction models. The inhibition of azithromycin on proinflammatory cytokines was described by an indirect response model. After lipopolysaccharide challenging, the proinflammatory cytokines, kynrenine and behavior tests would peak approximately at 3, 12, and 24 h respectively, and then the time courses slowly declined toward a baseline state after peak response. During azithromycin administration, the peak levels of proinflammatory cytokines, kynrenine and behavior indexes decreased. Model parameters indicated that azithromycin significantly inhibited the proinflammatory cytokines level in plasma and improved the depressive-like behavior induced by inflammation. The integrated model for disease progression and drug intervention captures turnovers of proinflammatory cytokines, kynrenine and the behavior results in the different time phases and conditions.

## Introduction

Increased major depressive-like disorders occur in many diseases (e.g., atherosclerosis, congestive heart failure, rheumatoid arthritis), all of which have a common inflammatory component [1]. For example, depressive-like behaviors were observed in patients undergoing cytokine immunotherapy for the treatment of cancers [2], [3]. In these conditions, depressive symptoms were induced by proinflammatory cytokines (PCs), mainly interleukin-1β (IL-1β), interleukin-6 (IL-6), interferon-γ (IFN-γ), and tumor necrosis factor-alpha (TNF-α) [4], [5].

Indoleamine 2,3-dioxygenase (IDO) is presented in macrophages and other cells that degrades tryptophan along the kynrenine (KYN) pathway. It was reported that IDO could be easily activated by PCs [6], [7], and its degree of activation (indicated by increased KYN concentration) was correlated to the intensity of depressive symptoms, as observed in cancer patients chronically treated with immunotherapy [7], [8]. The increased KYN concentration in circulating induced by PCs was potential to negatively impact serotoninergic neurotransmission in central nervous system (CNS) [6]. Meanwhile, KYN was readily transported across the blood brain barrier into the brain where it could be further metabolized by perivascular macrophages, microglia and astrocytes to generate neuroactive glutamatergic compounds [9], [10]. In fact, heightened glutamate activity might play an important role in major depression [11], [12]. Several studies evidenced that PCs-IDO -KYN -depressive-like behavior formed a complete relationship of inflammation-induced depressive-like behavior. Many papers reported that acute activation of the peripheral innate immune system in laboratory animals through the administration of the cytokine inducer lipopolysaccharide (LPS) induced depressive-like behavior, which could be attenuated by classical antidepressant administration in some treatments [13], [14]. While in order to test whether the activation of PCs by LPS was responsible for development of major depressive disorders, we investigated the depressive-like behavior after the inhibition of PCs in acute immune stimulation. Azithromycin (AZI) is one of the second generation macrolide antibiotics which is prepared semi-synthetically from erythromycin. AZI differs structurally from erythromycin by the presence of methyl-substituted nitrogen at position 9a in the macrolide ring. This modification leads to significant advantages for AZI, such as better stability in acidic pH [15], a longer half-life in serum, better tissue distribution with high peak levels, and a longer mean residence time [16], [17]. In the present study, AZI was chosen to reverse the PCs, because AZI has potent anti-inflammatory effects independent from its microbicidal properties, as it is well known to inhibit macrophage and microglial activation [18], [19]. We found that anti-inflammatory effects with AZI approach could abrogate LPS-induced depressive like behavior.

The pharmacokinetic-pharmacodynamic (PK-PD) model is a potential tool for enhancing the efficiency of decision-making in drug development [20]. PK-PD model-based approaches have been rapidly developed because of the insights they provide into how drugs exert their effects, thus improving the knowledge about drug processes and properties for extrapolation and prediction [21]. The PK-PD model has been widely applied in the field of CNS disorders and drugs [22], [23]. However, depressive-like behavior induced by PCs is often comorbid complication, which is different with classical depressive-like behavior. The present PK-PD model described the depressive-like behavior induced by PCs and antibiotics intervention, which distinguished from classical antidepressant. The PK-PD model of antibiotics was widely reported to investigate the relationship between the drug concentration and antibacterial effect (minimum inhibitory concentration, etc). However, the PK-PD model of antibiotics on disease progression of depressive-like disorders induced by inflammatory has not been investigated. In the present study, we assessed the potential effect of AZI on the progression of depressive-like behavior in LPS-challenge mice.

## Materials and Methods

### Animals

All animal care and use were conducted under a license granted by the Jiangsu Science and Technology Office (China) with approval from the Animal Ethics Committee of China Pharmaceutical University. Every effort was made to minimize the stress on the mice. Experiments were performed on 10–14 week-old male CD1 mice obtained from Vital River Laboratories (Beijing, China), whose average body weights were 35–40 g at the beginning of the experiments. Mice were individually housed in standard shoebox cages, with wood shavings litter, in a temperature (23°C) and humidity (45–55%) controlled environment with a 12/12-h modified dark-light cycle (light on 11∶00 PM–11∶00 AM). Food and water were available *ad libitum*. Mice were handled individually everyday for 10 days before the experiments.

### Chemical

LPS (L-3129, serotype 0127:B8) was purchased from Sigma (St. Louis, MO). AZI was purchased from Dawnrays Pharmaceutical Ltd (Suzhou, China). Other chemicals and solvents were purchased from Nanjing Chemical Reagent Co. Ltd (Nanjing, China).

### Study Design

On the day of injection, fresh solutions were prepared by dissolving LPS in sterile endotoxin-free isotonic saline and administered intraperitoneally (i.p.). The dose of LPS (0.8 mg/kg) was selected on the basis of its ability to induce the full spectrum of the acute sickness response and a reliable increase of IDO activity [24], a putative mechanism in the depressive-like behavior induced by LPS [25]. AZI was administered at an intragastric (i.g.) administration of 100 mg/kg on the same day 20 min prior to LPS injection. One set of animals was used for behavioral testing, including a locomotive activity, a forced swimming test (FST), and a tail suspension test (TST) (n = 8 per time point). In the other set of mice, plasma samples were collected at different time points for the analysis of AZI, LPS, PCs and KYN. The mice were sacrificed on each time point. (n = 8 per time point).

### LPS, PCs, KYN, and AZI Assays

#### LPS determination

LPS in plasma was measured by a commercially available tachypleus amebocyte lysate kit (Xiamen, China). Assays were sensitive with lower limits of quantification at 1 Eu/ml for LPS; internal intra-assay coefficients of variation were less than 10%.

#### PCs determination

IL-1β, IL-6, IFN-γ, and TNF-α in plasma were measured by commercially available enzyme linked immunosorbnent assay kits (Shanghai, China). Assays were sensitive with lower limits of quantification at 10 pg/ml for IL-1β, IL-6, IFN-γ, and TNF-α; Internal intra-assay coefficients of variation were less than 10%.

#### Determination of KYN in Plasma

Plasma KYN was analyzed by high performance liquid chromatography (HPLC) with an ultraviolet detector with λ = 360 nm. A reverse-phase C_18_ column was used. Mobile phase consisted of 15 mM sodium acetate-acetic acid buffer (pH  = 4.6): acetonitrile (v:v = 93∶3). Plasma was mixed with a solution of 10% perchloric acid solution (10 µl) and allowed to precipitate proteins on ice for at least 30 minutes. After the precipitation, samples were centrifuged at 12,000 × g for 10 minutes at 4°C. The supernatant was held at 4°C until a 20 µL volume was injected into the system. Standards were made by a serial dilution technique that made the standards to be the levels that would encompass expected levels in the plasma samples.

#### AZI determination

Blood AZI was measured by a previously reported HPLC-MS-MS method [26] with minor modifications. The quantification limit was 20 ng/ml for AZI with less than 10% internal intra-assay coefficients of variation.

### Behavioral Test

#### Locomotor activity

The assessment of locomotor activity was carried out on mice. Briefly, the locomotor activity of the mice was measured by an ambulometer with five activity chambers (Institute of Materia Medica, Chinese Academy of Medical Sciences, China). Mice were placed in the chambers and their paws contacted or disconnected the active bars producing random configurations that were converted into pulses. The pulses, which were proportional to the locomotor activity of the mice, were automatically recorded as the cumulative total counts of motor activity. Mice were placed in test chambers, 15 min prior to the evaluation for acclimatization and then locomotion counts were recorded for a period of 5 min.

#### FST activity

The FST was carried out as previously described with slight modifications [27]. Briefly, mice were placed individually in a clear cylinder (diameter 10 cm, height 25 cm), containing 15 cm of water at 25±1°C. The water was changed between testing sessions. Mice were forced to swim for 6 min, and the immobility time during the last 5 min was manually measured by a blinded observer. Mice were considered immobile when they ceased struggling, remained floating motionless, and only made those movements necessary to keep their head above the water [28].

#### TST activity

The TST was conducted as previously described [29]. Briefly, mice were suspended by adhesive tape that was positioned about 2.5 cm from the tail tap with the head 40 cm above the floor. The trial was carried out for 6 min and the duration of immobility was manually recorded by two blinded observers during the final 5 min interval of the test. Mice were considered immobile when they hung passively and motionlessly.

### AZI Pharmacokinetics

**Figure 1:**
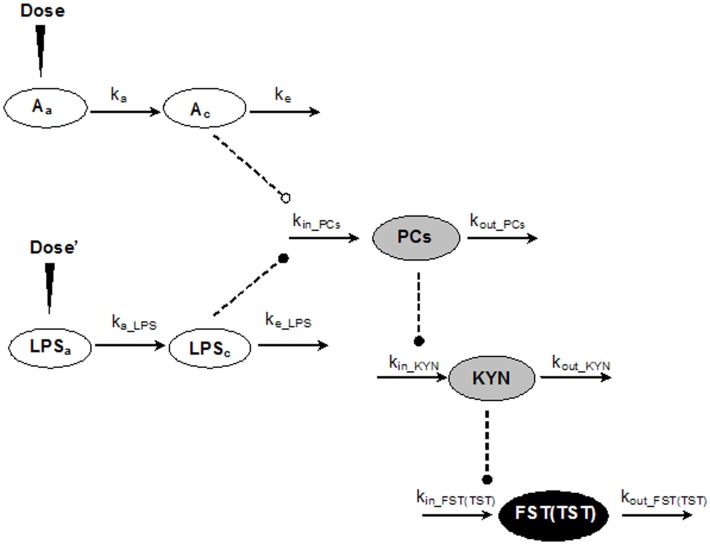
The schematic PK-PD model of the AZI intervention on the LPS -induced depressive-like behavior. Parameters and symbols were described in the text. Solid Lines with arrows indicated the disposition and turnover of the AZI, LPS, PCs, KYN and depressive-like behavior. Dashed lines with black dot meant the stimulatory effect being exerted by the connected factors. Dashed lines with white dot meant the inhibitory effect being exerted by the AZI. Long closed triangle symbols denoted the administration site for the AZI and LPS, respectively.

Fig. 1 showed a general schematic model for the entire PK-PD model. The PK of AZI was described by a one-compartment model with first-order absorption. The drug in the plasma was a driving force for the drug effect. The equations describing the amounts of AZI were as follows:
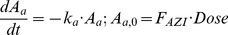
(1)

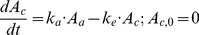
(2)
*A*_c_ and *A*_a_ were the amounts of the drug in the central and absorption compartment, respectively. *A*_c,0_, the initial amount of the drug in central compartment; *A*_a,0_, the initial amount of the drug in absorption compartment; *V*_AZI_ is used to estimate the distribution volume of drug. *k*_a_, the rate constant of the drug from absorption to central compartment; *k*_e_, the rate constant of the drug elimination from central compartment; F_AZI_, the drug fraction of absorption compartment.

### LPS-induced Depressive-like Model and AZI Pharmacodynamics

The animal model with depressive-like behavior induced by LPS in mice is a classical CNS disorder model that is characterized by increased LPS level, inflammation response [30], and KYN concentration in blood. LPS, PCs and KYN levels contributed to the onset of depressive-like behavior in mice. Based on these disease characteristics, LPS, PCs and KYN levels were considered as main disease components in LPS-induced depressive-like disease progression. The dispositions of LPS, PCs and KYN were characterized by transduction models, and the interactions among the three factors were described by indirect response models [31].

Because the LPS were injected i.p., the LPS was assumed to diffuse into circulation by a first-order constant. The disposition of LPS was described by:
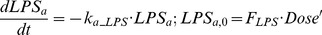
(3)

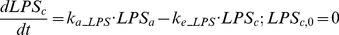
(4)LPS_a_ and LPS_c_ were the amounts of LPS in the abdominal cavity and circulation, respectively. LPS_a,0_ and LPS_c,0_ were the initial amounts of LPS in the abdominal cavity and circulation. The LPS concentration was calculated by the hypothetical distribution volume of LPS (V_LPS,_ per nanogram LPS equal to 3.4 Eu approximately). *k*_a_LPS_, the rate constant of the LPS from absorption to central compartment; *k*_e_LPS_, the rate constant of the LPS elimination from central compartment; F_LPS_, the LPS fraction of absorption compartment.

The LPS in circulation stimulated the specific antigen in microphone cell to induce the production of PCs. Successively, the PCs, mainly IL-1β, IL-6, IFN-γand TNF-α, activated the IDO in peripheral tissue and increased KYN level in blood. The two transduction models were used to describe the turnovers of PCs and KYN. After AZI administration, the production of PCs was inhibited effectively rather than the elimination [32]. Thus, a time-dependent indirect inhibitory function was used to describe the inhibition of production of PCs [33]. The inhibitory function of AZI on the production of PCs was appeared as follows:
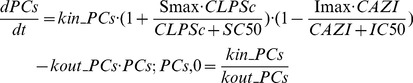
(5)

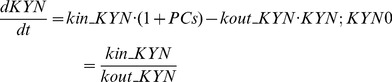
(6)


As a PD end point, the depressive-like behavior (FST or TST) was link to peripheral KYN level as the followed:
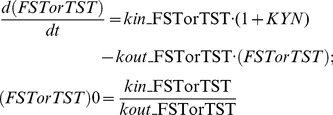
(7)


### Computation of PK-PD Model

The proposed LPS-induced depressive model and AZI pharmacodynamics was constructed in two phases. For the PK phase, non-compartmental analysis by Excel software (Microsoft, Redmond, WA) was first performed for the initial estimated compartmental PK parameters. Fitting of the compartmental PK parameters for AZI was performed using equations 1–2.

LPS-induced depressive model was depicted by several transduction compartments of endogenous substances disposition. In the PD model of AZI, the PD was expressed by an indirect response model. The compartmental PD parameters were estimated by the fully integrated model described by equations 3–7. All parameters were estimated by ADAPT II software [34]. The program code of differential functions was showed in appendix (Appendix S1). The naïve pool method and bootstrap analysis was used to estimate the model parameters. Individual data were used to estimate the PK-PD parameters by naive pooling approach, which is by maximizing the sum of likelihood (like putting more weight on small values). This method is more advanced in handling large range of values and variability. The goodness-of-fit was assessed by Akaike’s information criterion, examination of residuals, and visual inspection.

### Data Analysis

Data were expressed as the mean ± coefficient of variation (CV%). Differences between multiple groups were evaluated by Student’s *t*-test using SPSS software. Differences were considered statistically significant at *P*<0.05.

## Results

### AZI Pharmacokinetics

**Figure 2:**
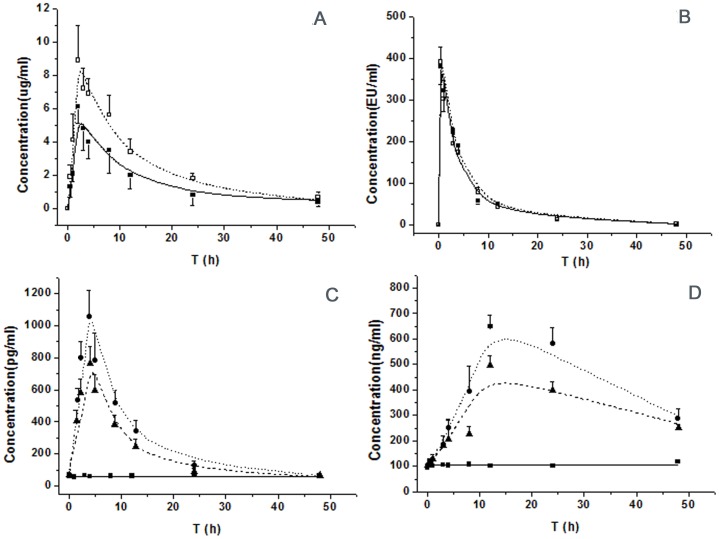
Concentration-time profiles of AZI (A), LPS (B), total PCs (C), and KYN(D) after an i.p. LPS (0.8 mg/kg) and an i.g. AZI (100 mg/kg) in mice. (A): Closed squares and open squares indicated measured values in normal and LPS loading mice, respectively. Model fittings were presented as solid line for the normal mice and dash line for the LPS loading mice, respectively. (B): Closed squares and open squares indicated measured values in normal and AZI-treated mice, respectively. Model fittings were presented as solid line for normal mice and dash line for AZI-treated mice, respectively. (C) and (D): control mice were shown in closed squares and solid line. The model group was shown in closed circles and dot line. The regulative effect of AZI was shown in closed triangle with dash line. All observations were reported as Mean ±SD (n = 8) in Figure 2.

Table 1 showed the one-compartmental PK parameters of AZI in normal and LPS-challenging mice. The terminal half-lifes of AZI in two groups were 17.3 h and 16.5 h respectively. Compared to normal mice, the areas under the concentration-time curves (AUC_0–∞_) increased from 81.7 to 143.6 ug·h/mL, and the peak levels of AZI elevated from 6.1 to 8.9 ug/mL at 2 h. Fig. 2A showed the time-course of AZI levels in plasma after i.g. administration of AZI (100 mg/kg). The predicted plasma concentration curve was well fitted by a one-compartment PK model and estimated parameters.

**Table 1:** The Pharmacokinetic Parameters of Azithromycin in Normal Mice and LPS-Challenging Mice.

Parameter (unit)	Definition	Normal Mice		LPS-Challenging Mice	
		Estimated value	CV%	Estimated value	CV%
k_a_ (h^−1^)	The absorption rate constant of azithromycin	0.78	43	0.85	40
k_e_ (h^−1^)	The elimination rate constant of azithromycin	0.040	38	0.042	33
V_AZI_/F_AZI_ (L/kg)	Distribution volume of azithromycin	42.1	35	28.5	36

### LPS Pharmacokinetics

To minic a state that gram-negative bacterial was killed and the LPS was released from the wall of gram-negative bacterial, we injected the LPS to challenge the LPS- PCs -KYN-CNS disorder. No PK difference was observed in normal and AZI-treating mice as shown in table 2. The terminal half-life of LPS in mice were 8.4 and 7.8 h respectively, and the AUC_0–∞_ were 2315.2 and 2247.6 Eu·h/mL respectively in normal and LPS-challenge mice. The measured and predicted LPS concentrations in two groups in plasma were shown in Fig. 2B.

**Table 2:** The Pharmacokinetic Parameters of LPS in Normal Mice and Azithromycin-Treating Mice.

Parameter (unit)	Definition	Normal Mice		Azithromycin-Treating Mice	
		Estimated value	CV(%)	Estimated value	CV(%)
k_a_LPS_ (h^−1^)	The absorption rate constant of LPS	1.51	39	1.63	37
k_e_LPS_ (h^−1^)	The elimination rate constant of LPS	0.082	34	0.088	33
V_LPS_/F_LPS_ (L/kg)	Distribution volume of LPS	16.3	32	15.5	36

### LPS Induced-depressive Model

**Figure 3:**
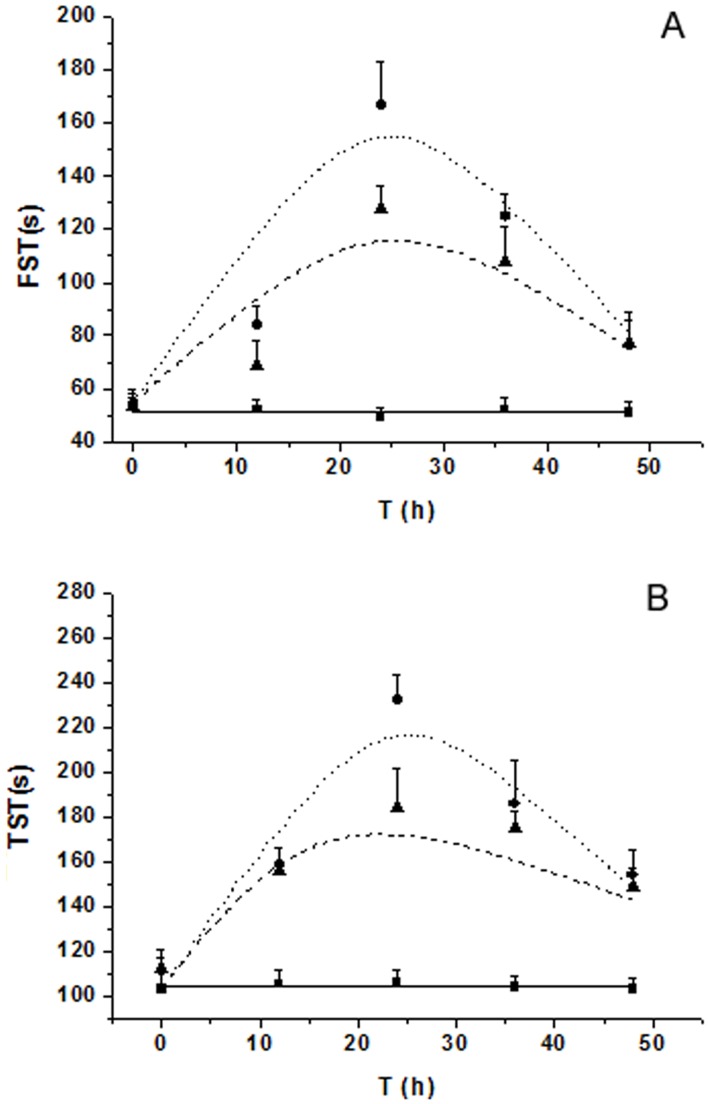
The behavioral profiles of FST (A) and TST (B) after an i.p. LPS (0.8 mg/kg) and AZI treatment (100 mg/kg) in mice. Measured values and model fittings were shown in symbols and lines, respectively. Control mice were shown in closed squares and solid line. The model group was shown in closed circles and dot line. The regulative effect following a single oral dose of AZI was shown in closed triangle with dash line. All observations were reported as Mean ±SD (n = 8).

No obvious change of PCs, KYN level and depressive-like behavior was observed in control mice. In the group of AZI only, the time courses of PCs, KYN level and depressive-like behavior were similar to the control mice, so the data were not shown in results. In Fig. 2C, the total PCs concentration was used to model PK-PD parameters. The total peak concentration of PCs at 3 h was 1055 pg/ml after an i.p. LPS injection, (including IL-1β214 pg/ml, IL-6 287 pg/ml, IFN-γ176 pg/ml, and TNF-α 378 pg/ml). After AZI intervention, the increased PCs induced by LPS could be reversed partially. The peak concentration at 3 h decreased to 762 pg/ml, (including IL-1 β135 pg/ml, IL-6 202 pg/ml, IFN-γ 126 pg/ml, and TNF-α 299 pg/ml). As shown in Fig. 2D, the peak time of KYN challenged by LPS appeared at 12 h with an upsurge of 649 ng/ml. In AZI treatment group, the KYN concentration significantly attenuated similar to the total PCs (*p*<0.05). The depressive-like behavior in CNS including FST and TST were shown in Fig. 3. The peak time of FST and TST were appeared in 24 h, and the depressive-like behavior still had not returned to the baseline until 48 h. The observed profiles of PCs, KYN level and CNS behavior were reasonably captured by the LPS-induced depressive models and AZI pharmacodynamics.

Final estimated PD parameters and coefficient of variation were summarized in Table 3. We formulated for describing the different disease elements in equation 3–7. PCs,_0_ and k_out_PCs_ were estimated to 49.8 pg·ml^−1^and 0.234 h^−1^; KYN_0_ and k_out_KYN_ were 104.3 ng·ml^−1^ and 0.167 h^−1^. As PD endpoint, the turnover of depressive-like behavior was also be described as a hypothetical input and output rate though the CNS behavior was not a substantial endogenous substance. The FST_0_ and k_out_FST_ were 51.3 s and 0.253 h^−1^, and the TST_0_ and k_out_TST_ were 108.2 s and 0.143 h^−1^. The predictive performance of the model, as shown in Fig. 2 and Fig. 3, was reasonable and adequately reflected the trend and variability of the raw data. The ameliorative effect of AZI on the occurrence of PCs and subsequent results were sufficiently described by the indirect inhibitory function. The suppressive fractions of AZI on PCs were 6.3 ug/ml (IC_50_) and 0.83 (I_max_). As an inducer, the infinity and efficacy of LPS on PCs were also calculated, the estimated SC_50_ and S_max_ were 103.5 Eu/ml and 27.5 for LPS, which showed that the LPS had a high capacity and power to challenge the production of PCs.

**Table 3:** Parameter Estimates for the LPS-Induced Depressive like Model and Azithromycin Pharmacodynamics.

Parameter (units)	Definition	Estimated value	CV%
PCs,_0_ (pg·ml^−1^)	Initial value of plasma proinflammatory cytokines	49.8	35
k_out_PCs_ (h^−1^)	Elimination rate constant of plasma proinflammatory cytokines	0.234	38
KYN_0_ (ng·ml^−1^)	Initial value of plasma kynurenine	104.3	31
k_out_KYN_ (h^−1^)	Elimination rate constant of plasma kynurenine	0.167	35
FST_0_ (TST_0_) (s)	Initial value of forced swimming test (tail suspension test)	51.3 (108.2)	34 (41)
k_out_ FST(TST)_ (h^−1^)	Hypothetical elimination rate constant of forced swimming test(tail suspension test)	0.253(0.143)	33 (35)
I_max_	Capacity factor for azithromycin induced inhibition on proinflammatory cytokines	0.83	34
IC_50_ (ug/ml)	Concentration of azithromycin producing 50% I_max_	6.3	31
S_max_	Capacity factor for LPS induced stimulation on proinflammatory cytokines	27.5	42
SC_50_(EU/ml)	Concentration of LPS producing 50% S_max_	103.5	39

## Discussion

Depression is a worldwide problem for humans, and commonly selective serotonin reuptake inhibitors and monoamine oxidase inhibitors were used to improve depressive-like behavior [35]. However, their therapeutic effects only manifest in 28%–63% of depressed patients [36]. The classic monoamine hypothesis of depression has been challenged. Accumulating evidences reveals a close linkage between inflammation and depression. Depressive symptoms frequently develop in chronically infected patients accompanied with an increase of PCs [37]. In recent investigation, it has been suggested that inflammation-induced IDO activation resulted in an accumulation of tryptophan metabolite, KYN, ultimately leading to the development of depression [38].

The stimulation of innate immune system and subsequent release of PCs in peripheral by i.p. LPS may also cause neuroinflammatory in CNS through both direct (humoral) and indirect (neural) pathway to relay peripheral inflammatory signal to the CNS [39]. A small part of PCs in blood induced by i.p. LPS can gain access through relatively permeable areas of the blood-brain barrier [40]. On the other way, activation of the vagal nerve afferent pathway may also account for CNS inflammation [41]. The neuroinflammation has also activated the IDO in neuronal cells and glial cells and increased brain KYN level. However, the equilibrium percentage of KYN in the brain originated from KYN in the plasma was 78±6% in control mice, while it was 60% in normal rats [42]. The plasma contribution rose to 100±19% with i.p. LPS systemic activation [43]. From these results, the increased KYN level in brain was exclusively derived from the transport of blood KYN level after i.p. LPS, rather than from the synthesis of IDO in brain.

The delay appeared in the time courses among LPS, PCs, KYN, and depressive-like behavior. The different molecular mechanisms contributed to the lag time of multiple PD surrogates and endpoints. It is well known that LPS binds to a LPS binding protein in blood, and the interaction of LPS- LPS binding protein with its receptor triggers the monocytic secretion of several PCs [44]. Some reports have recently shown that the PCs-induced increase of IDO activity was associated with an increased transcription of IDO mRNA. Pharmacological data also showed that the delayed KYN increase induced by synergistic effect of IL-1β, IL-6, IFN-γ and TNF-α were mediated by IDO activation [45]. KYN and KYN metabolites such as quinolinic acid and 3-hydroxykynurenine in brain, which were known to be neurotoxic and thereby might lead to depression-like behaviors through both serotonin and glutamate pathways [46], [47].

Macrolide antibiotics are a well established class of antibacterial agents those are active against many gram-positive and gram-negative bacteria. Beyond their antibacterial activity, macrolide antibiotics are reported to exert anti-inflammatory and immunomodulatory activity in vitro and in vivo [48]. It has been reported previously that macrolide antibiotics can affect several steps in the inflammatory process, such as migration of neutrophils, modulation of oxidative burst and production of cytokines [48], [49]. AZI is a new broad-spectrum, second-generation macrolide antibiotic for clinial use, which has been widely used to treat a broad array of infectious diseases such as pneumonia, peritonitis and mastitis [50]. AZI reduced the production of PCs, like IL-1ß and TNF-α, in response to LPS stimulation, which was responsible for the clinical effectiveness in chronic inflammatory disorders [50], [51]. In patients, the LPS was released from the wall of gram-negative bacterial, and occurred in blood when these bacterial were killed by antibacterial agents, so the application of antibacterial agents were double-edged sword to limit the use of antibacterial agents in clinic. In the present study, the administration of LPS miniced the pathological process of the death of bacteria and the release of LPS from bacteria after AZI administration in systemic bacterial infection. In previous research, minocycline and tetracycline were used to relieve the burst of PCs and improve the depressive-like behavior. Minocycline was regard as an antibiotic with anti-inflammatory action against the PCs after i.p. injection of LPS, which has the great permeability through the blood brain barrier. Consequently, the anti-inflammatory effect of minocycline on CNS and periphery has commonly contributed to the benefit of minocycline on CNS disorder [52]. In the present study, AZI showed a similar effect with minocycline. However, AZI had a weak penetration to distribute from blood to brain, so the benefit effect of AZI on CNS disorder was mainly derived from the effect of AZI on peripheral anti-inflammatory action. Compared to the normal mice, the AZI exposure in LPS loading mice has a significant increase as Fig. 2A. After an i.p. injection of LPS, the inflammation was induced by LPS in peripheral, especially in intestinal tract. The increased inflammatory factor and inflammatory reaction induced by LPS would damage the structure of the biological membrane and increase the permeability of intestinal tract. The increased permeability of intestinal tract was benefit for the intestinal microvascular transports of AZI and improved the absorption of AZI after an i.g. administration.

The results in present study could be used to describe the response-concentration-time relationships of AZI by a mathematical model and parameters as opposed to concentration profiles. The estimated PK-PD parameters were based on data from single dose levels of AZI. The large perturbation will make a full change of biological system (mostly nonlinear) and allow a clear characterization of dose-response relationship. Generally, a wide range of dose will benefit model prediction and high dose is essential for capturing the nonlinear system. Thus, our study designed a very high dose of AZI to support a PKPD characterization. As it is mentioned in eq. 5, the inhibitory function of AZI and the stimulatory function of LPS on the production of PCs were appeared in one equation. Although two functions were included in eq. 5, the only-LPS experimental group (disease control) would allow us to specifically evaluate the effect of LPS on PC production (Smax and SC50). Additionally, the opposite directions of the two functions and distinct kinetics of LPS and AZI would give different influence to PC production, which also gave us possibilities to separately evaluate these two functions. In equation 5, we used Sigmoid model to describe both the stimulation of LPS and the inhibition of AZI on production of PCs, while used linear model to describe the stimulation of PCs on production of KYN (eq. 6) and he stimulation of KYN on production of FST or TST(eq. 7). Generally sigmoid model would be better than linear model to characterize biological functions, but the final model was chosen based upon several model fitting criteria, such as parameters precision, and visual checking. The linear model could parsimoniously capture the data with reasonable precision of estimates. Additionally, the linear model that used in equation 6 and 7 reduced the estimated parameters and simplified the structure in the final model, because we mainly focused on the processes of the stimulation of LPS and the inhibition of AZI in this article. The Akaike’s information criterion of the final model of LPS and AZI intervention is 22.31. The residual errors of the AZI, LPS, PCs, KYN and behavioral test (FST and TST) are all less than 38%. Initially, various numbers of transit compartments were tested to describe the production and elimination process of PCs, KYN and CNS disorder. Suitable parameters (SC_50_, IC_50_, S_max_, I_max_, k_in_, k_out_) were obtained by classical indirect response model and one transfer compartment, demonstrating that this was sufficient to account for the production and elimination different physiological processes.

A number of PK-PD modeling efforts have been reported for describing the inflammation [53], endogenous amino acid neurotransmitters [54], and CNS disorder disease progression in animal and human separately [55]. In our study, the present PK-PD model was introduced to investigate the interaction of LPS-PCs-KYN-CNS disorder, as well as incorporating AZI treatment to evaluate the potential involvement of inflammation on depressive progression.

## Supporting Information

Appendix S1 **The program code of differential functions in ADAPT II software.**
(DOC)
