# Supplementary material for: The Pharmacokinetic-Pharmacodynamic Model of Azithromycin for Lipopolysaccharide-Induced Depressive-Like Behavior in Mice
Source: PLoS One. 2013 Jan 24;8(1):e54981. doi: 10.1371/journal.pone.0054981 (PMC3554664; doi:10.1371/journal.pone.0054981)
Supplement: Appendix S1 — The program code of differential functions in ADAPT II software. (DOC) [file pone.0054981.s001.doc]

**Supporting Information**

**Legend: Appendix S1 The program code of differential functions in ADAPT II software.**

The corresponding differential code to the equation 1-7 in article as follows:

$XP(1)=-P(1)*X(1)$ (1)

$XP(2)=P(1)*X(1)-P(2)*X(2)$ (2)

$XP(3)=-P(3)*X(3)$ (3)

$XP(4)=P(3)*X(3)-P(4)*X(4)$ (4)

$XP(5)=P(5)*(1+\frac{P(6)*X(4)/P(7)}{X(4)/P(7)+P(8)})*(1-\frac{P(9)*X(2)/P(10)}{X(2)/P(10)+P(11)})-P(12)*X(5)$ (5)

$XP(6)=P(13)*(1+X(5))-P(14)*X(6)$ (6)

$XP(7)=P(15)*(1+X(6))-P(16)*X(7)$ (7)

The output code to the equation 1-7 in article as follows:

$$Y(1)=X(2)/P(10)$$

$$Y(2)=X(4)/P(7)$$

$$Y(3)=X(5)$$

$$Y(4)=X(6)$$

$$Y(5)=X(7)$$
